# A quantitative genetic model for indirect genetic effects and genomic imprinting under random and assortative mating

**DOI:** 10.1093/genetics/iyag042

**Published:** 2026-02-12

**Authors:** Ilse Krätschmer, Matthew R Robinson

**Affiliations:** Institute of Science and Technology Austria, Klosterneuburg 3400, Austria; Institute of Science and Technology Austria, Klosterneuburg 3400, Austria

**Keywords:** quantitative genetics model, epigenetic, parent-of-origin, indirect effects

## Abstract

An individual’s phenotype reflects a complex interplay of the direct effects of their DNA, epigenetic modifications of their DNA induced by their parents, and indirect effects of their parents’ DNA. Here, we derive how the genetic variance within a population is changed under the influence of indirect maternal, paternal, and parent-of-origin effects under random mating. We also consider indirect effects of a sibling, in particular how the genetic variance is altered when looking at the phenotypic difference between two siblings. The calculations are then extended to include assortative mating (AM), which alters the variance by inducing increased homozygosity and correlations within and across loci. AM likely leads to covariance of parental genetic effects, a measure of the similarity of parents in the indirect effects they have on their children. We propose that this assortment for parental characteristics, where biological parents create similar environments for their children, can create shared parental effects across traits and the appearance of cross-trait AM. Our theory shows how the resemblance among relatives increases under both AM, indirect, and parent-of-origin effects. When our model is used to predict correlations among relatives in human height, we find that explaining the patterns observed in real data requires both indirect genetic effects and AM. The degree to which direct, indirect, and epigenetic effects shape the phenotypic variance of complex traits remains an open question that requires large-scale family data to be resolved.

## Introduction

**Fig. 1.**
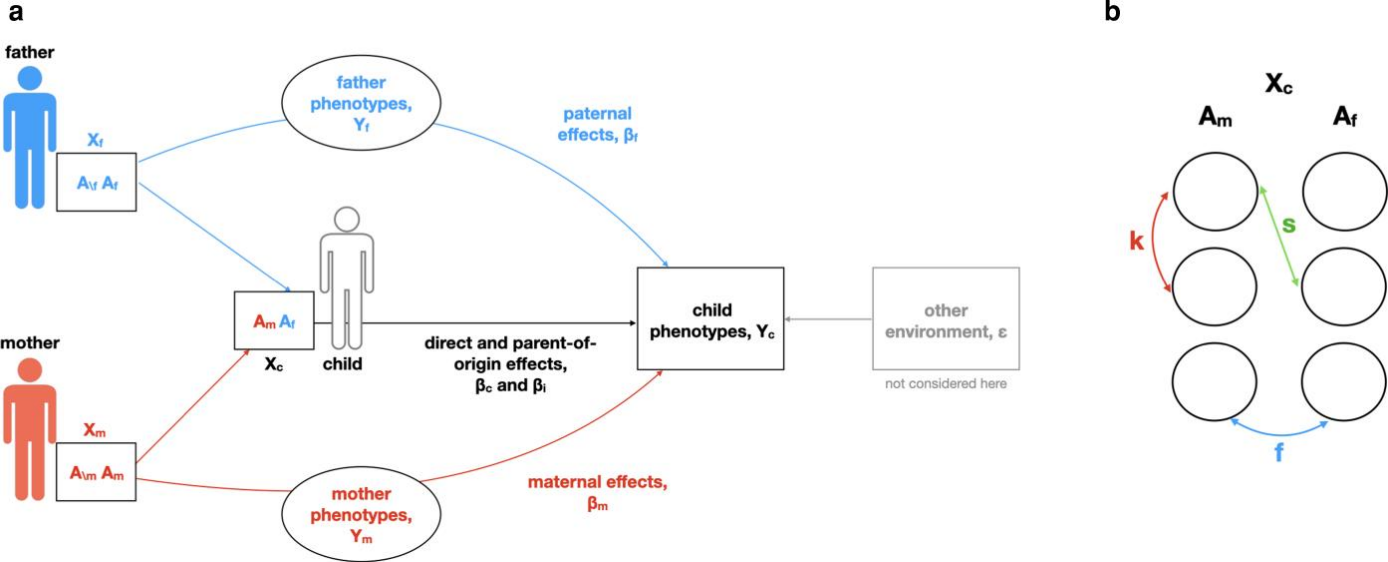
Definition of variables. a) Direct ($\beta_{c}$), parent-of-origin ($\beta_{i}$), and indirect maternal ($\beta_{m}$) and paternal ($\beta_{f}$) genetic effects on the phenotypes of children ($Y_{c}$). The parental genotypes ($X_{m}$, $X_{f}$) are split into transmitted alleles ($A_{m}$, $A_{\;f}$), which constitute the genotype of the child ($X_{c}$), and untransmitted ones ($A_{\setminus m}$, $A_{\setminus f}$). Parental effects can be mediated through any parental phenotype ($Y_{m}$, $Y_{\;f}$). The variable ϵ represents variations created by other environmental effects experienced by the children. b) Correlations within and between loci introduced through AM. The child’s DNA ($X_{c}$) consists of maternally inherited alleles ($A_{m}$) and paternally inherited ones ($A_{f}$). The correlation between alleles at a locus is given by *f*, the one between alleles at different loci within the same gamete by *k* and the one between pairs of $A_{m}$ and $A_{f}$ alleles at different loci by *s*.

In humans, parental influence persists long beyond childhood, shaping adult health and disease (Gluckman et al. 2008 ). A basic principle of genetics is that observable characteristics result from two sources: the expression of an individual’s DNA and the environment that they experience (Lynch and Walsh 1998). Parental characteristics shape a child’s environment, giving rise to indirect genetic effects, where the genotypes of both the mother and father influence the traits of their children, beyond simply the alleles that are inherited (Lynch and Walsh 1998), as displayed in Fig. 1. Many studies have now shown that failing to separate the effects of an individual’s DNA from the indirect effects of their parental genotypes results in confounding within genetic association studies (Kong et al. 2018; Shen and Feldman 2020; Wolf 2000; Eilertsen et al. 2021). Moreover, it has been shown that maternal effects cause the appearance of parent-of-origin effects that can mimic genomic imprinting, and vice versa (Hager et al. 2008). Parent-of-origin effects arise when the effect of an allele depends on its parental origin.

Previous quantitative genetics models derived the genetic population variance under the influence of maternal effects (Wolf 2000; Wolf and Wade 2016), or imprinting (Spencer 2002) or both (Santure and Spencer 2006; Wolf and Hager 2006; Hager et al. 2008) under random mating (RM). Wolf et al. demonstrated how indirect maternal effects contribute to the variance under inbreeding (Wolf and Wade 2016). Other studies (Veller et al. 2024; Veller and Coop 2024; Young 2023) provided a general framework to show the influence of confounding in family-based genome-wide association studies (GWAS), and how polygenic risk scores are biased under assortative mating (AM) in the presence of indirect parental effects.

But so far, none of the models considered direct, indirect maternal and paternal, and parent-of-origin genetic effects jointly. Here, we first derive the genetic variance for a single locus under RM assuming that all four effects (direct, indirect maternal and paternal, and parent-of-origin) influence an individual’s phenotype. We then extend the model to include also the indirect effects of a sibling and consider another common family-based design in human studies: the phenotypic difference between two siblings. Next, we show how the genetic variance changes in the presence of AM (or inbreeding). Lastly, we demonstrate how to include multiple loci in the model. We then also verify our theoretical model with forward-in-time simulations, based on genotypes from the 1000 Genomes project (Auton et al. 2015). Finally, we compare our theoretical predictions for phenotypic correlation as function of genomic relatedness against real data from the UK Biobank given in Kemper et al. (2021).

Though we primarily consider humans and use the corresponding terms, we want to point out that our calculations are not limited to humans but generally applicable to diploid sexually reproducing species that rear their offspring.

## Single-locus model under random mating

We first consider a simple single autosomal locus with two alleles ($A_{1}$ and $A_{2}$) with frequencies $q_{1}$ and $q_{2}$ that can have the following four effects on the child’s phenotype: (i) direct ($\beta_{c}$), which are the effects that a child’s genotype has on the child’s phenotype; (ii) indirect maternal ($\beta_{m}$) and (iii) indirect paternal ($\beta_{f}$), which correspond to the effects that the mother’s and accordingly father’s genotype has on the child’s phenotype and which are mediated through the maternal and paternal phenotypes, $Y_{m}$ and $Y_{f}$; and (iv) parent-of-origin (imprinting, $\beta_{i}$), which reflects whether the allele $A_{2}$ was inherited from the mother or the father in case of heterozygous loci. The alleles of the child at the locus are ordered, referring first to the maternally inherited allele and second to the paternally inherited one, i.e. $A_{m}A_{f}$. The genotypes $X_{c}$, $X_{m}$, $X_{f}$, are coded as 0, 1, and 2 for $A_{1}A_{1}$ homozygotes, $A_{1}A_{2}$ or $A_{2}A_{1}$ heterozygotes and $A_{2}A_{2}$ homozygotes, respectively. We use subscripts m, f, c, and i to denote maternal, paternal, direct (child) and parent-of-origin (imprinting) effects, genotypes, and genotypic values. For heterozygous children, the parent-of-origin effect is positive for maternally inherited $A_{2}$ and negative for paternally inherited $A_{2}$. From these definitions, the possible genotypes, $X_{c}$, and phenotypes, $Y_{c}$, of the child given the genotype of the parents and imprinting, are shown in Table 1. The phenotypic values represent the deviations from the case where both parents and child have $A_{1}A_{1}$ genotypes.

**Table 1.** A simple single-locus model with indirect parental and parent-of-origin genetic effects.

$X_{m}$	$X_{f}$	$X_{c}$ $\left(A_{m}A_{f}\right)$	$Y_{c}$				z	$z_{\mathrm{AM}}$
$A_{1}A_{1}$	$A_{1}A_{1}$	$A_{1}A_{1}$					$q_{1}^{4}$	$\left[q_{1}^{4}+2\rho q_{1}^{3}q_{2}+\rho^{2}q_{1}^{2}q_{2}^{2}][1+\frac{\rho }{S}4q_{2}^{2}\right]$
	$A_{1}A_{2}$	$A_{1}A_{1}$			$+\beta_{f}$		$q_{1}^{3}q_{2}$	$\left[q_{1}^{3}q_{2}+\rho q_{1}^{2}q_{2}(q_{2}-q_{1})-\rho^{2}q_{1}^{2}q_{2}^{2}][1+\frac{\rho }{S}2q_{2}(q_{2}-q_{1})\right]$
		$A_{1}A_{2}$	$+\beta_{c}$		$+\beta_{f}$	$-\beta_{i}$	$q_{1}^{3}q_{2}$	$\left[q_{1}^{3}q_{2}+\rho q_{1}^{2}q_{2}(q_{2}-q_{1})-\rho^{2}q_{1}^{2}q_{2}^{2}][1+\frac{\rho }{S}2q_{2}(q_{2}-q_{1})\right]$
	$A_{2}A_{2}$	$A_{1}A_{2}$	$+\beta_{c}$		$+2\beta_{f}$	$-\beta_{i}$	$q_{1}^{2}q_{2}^{2}$	$\left[q_{1}^{2}q_{2}^{2}+\rho q_{1}q_{2}(q_{1}^{2}+q_{2}^{2})+\rho^{2}q_{1}^{2}q_{2}^{2}][1+\frac{\rho }{S}(-4q_{1}q_{2})\right]$
$A_{1}A_{2}$	$A_{1}A_{1}$	$A_{1}A_{1}$		$+\beta_{m}$			$q_{1}^{3}q_{2}$	$\left[q_{1}^{3}q_{2}+\rho q_{1}^{2}q_{2}(q_{2}-q_{1})-\rho^{2}q_{1}^{2}q_{2}^{2}][1+\frac{\rho }{S}2q_{2}(q_{2}-q_{1})\right]$
		$A_{2}A_{1}$	$+\beta_{c}$	$+\beta_{m}$		$+\beta_{i}$	$q_{1}^{3}q_{2}$	$\left[q_{1}^{3}q_{2}+\rho q_{1}^{2}q_{2}(q_{2}-q_{1})-\rho^{2}q_{1}^{2}q_{2}^{2}][1+\frac{\rho }{S}2q_{2}(q_{2}-q_{1})\right]$
	$A_{1}A_{2}$	$A_{1}A_{1}$		$+\beta_{m}$	$+\beta_{f}$		$q_{1}^{2}q_{2}^{2}$	$\left[q_{1}^{2}q_{2}^{2}-2\rho q_{1}^{2}q_{2}^{2}+\rho^{2}q_{1}^{2}q_{2}^{2}][1+\frac{\rho }{S}(q_{1}-q_{2}{)}^{2}\right]$
		$A_{2}A_{1}$	$+\beta_{c}$	$+\beta_{m}$	$+\beta_{f}$	$+\beta_{i}$	$q_{1}^{2}q_{2}^{2}$	$\left[q_{1}^{2}q_{2}^{2}-2\rho q_{1}^{2}q_{2}^{2}+\rho^{2}q_{1}^{2}q_{2}^{2}][1+\frac{\rho }{S}(q_{1}-q_{2}{)}^{2}\right]$
		$A_{1}A_{2}$	$+\beta_{c}$	$+\beta_{m}$	$+\beta_{f}$	$-\beta_{i}$	$q_{1}^{2}q_{2}^{2}$	$\left[q_{1}^{2}q_{2}^{2}-2\rho q_{1}^{2}q_{2}^{2}+\rho^{2}q_{1}^{2}q_{2}^{2}][1+\frac{\rho }{S}(q_{1}-q_{2}{)}^{2}\right]$
		$A_{2}A_{2}$	$+2\beta_{c}$	$+\beta_{m}$	$+\beta_{f}$		$q_{1}^{2}q_{2}^{2}$	$\left[q_{1}^{2}q_{2}^{2}-2\rho q_{1}^{2}q_{2}^{2}+\rho^{2}q_{1}^{2}q_{2}^{2}][1+\frac{\rho }{S}(q_{1}-q_{2}{)}^{2}\right]$
	$A_{2}A_{2}$	$A_{1}A_{2}$	$+\beta_{c}$	$+\beta_{m}$	$+2\beta_{f}$	$-\beta_{i}$	$q_{1}q_{2}^{3}$	$\left[q_{1}q_{2}^{3}+\rho q_{1}q_{2}^{2}(q_{1}-q_{2})-\rho^{2}q_{1}^{2}q_{2}^{2}][1+\frac{\rho }{S}2q_{1}(q_{1}-q_{2})\right]$
		$A_{2}A_{2}$	$+2\beta_{c}$	$+\beta_{m}$	$+2\beta_{f}$		$q_{1}q_{2}^{3}$	$\left[q_{1}q_{2}^{3}+\rho q_{1}q_{2}^{2}(q_{1}-q_{2})-\rho^{2}q_{1}^{2}q_{2}^{2}][1+\frac{\rho }{S}2q_{1}(q_{1}-q_{2})\right]$
$A_{2}A_{2}$	$A_{1}A_{1}$	$A_{2}A_{1}$	$+\beta_{c}$	$+2\beta_{m}$		$+\beta_{i}$	$q_{1}^{2}q_{2}^{2}$	$\left[q_{1}^{2}q_{2}^{2}+\rho q_{1}q_{2}(q_{1}^{2}+q_{2}^{2})+\rho^{2}q_{1}^{2}q_{2}^{2}][1+\frac{\rho }{S}(-4q_{1}q_{2})\right]$
	$A_{1}A_{2}$	$A_{2}A_{1}$	$+\beta_{c}$	$+2\beta_{m}$	$+\beta_{f}$	$+\beta_{i}$	$q_{1}q_{2}^{3}$	$\left[q_{1}q_{2}^{3}+\rho q_{1}q_{2}^{2}(q_{1}-q_{2})-\rho^{2}q_{1}^{2}q_{2}^{2}][1+\frac{\rho }{S}2q_{1}(q_{1}-q_{2})\right]$
		$A_{2}A_{2}$	$+2\beta_{c}$	$+2\beta_{m}$	$+\beta_{f}$		$q_{1}q_{2}^{3}$	$\left[q_{1}q_{2}^{3}+\rho q_{1}q_{2}^{2}(q_{1}-q_{2})-\rho^{2}q_{1}^{2}q_{2}^{2}][1+\frac{\rho }{S}2q_{1}(q_{1}-q_{2})\right]$
	$A_{2}A_{2}$	$A_{2}A_{2}$	$+2\beta_{c}$	$+2\beta_{m}$	$+2\beta_{f}$		$q_{2}^{4}$	$\left[q_{2}^{4}+2\rho q_{1}q_{2}^{3}+\rho^{2}q_{1}^{2}q_{2}^{2}][1+\frac{\rho }{S}4q_{1}^{2}\right]$

Maternal ($X_{m}$), paternal ($X_{f}$) and child’s genotypes ($X_{c}$), phenotypes of the child ($Y_{c}$), mating frequencies under RM (*z*) and under AM ($z_{\mathrm{AM}}$) taken from Stark (1976) where $S=2q_{1}q_{2}(1+\rho )$. Parental heterozygous genotypes are not ordered and are used interchangeably. Refer to text for more details.

Table 1 shows that indirect parental genetic effect genotypic values, $X_{m}\beta_{m}$ and $X_{f}\beta_{f}$, and parent-of-origin effect genotypic values, $X_{i}\beta_{i}$, are confounded due to the fact that not every child genotype can be produced by all parental genotypes. For example, parents who are both the same type of homozygote (both either $A_{1}A_{1}$ or $A_{2}A_{2}$) will only have $A_{1}A_{1}$ and $A_{2}A_{2}$ children. Each of the two types of homozygous parents ($A_{1}A_{1}$ and $A_{2}A_{2}$) can produce only one of the two types of reciprocal heterozygote ($A_{1}A_{2}$ and $A_{2}A_{1}$). The children of $A_{1}A_{1}$ fathers all have the term $+\beta_{i}$ as part of their phenotypes, $Y_{c}$, with the exception of $A_{1}A_{1}$ children whose parents are both $A_{1}A_{1}$. The children of $A_{2}A_{2}$ fathers all have the term $-\beta_{i}$, with the exception of $A_{2}A_{2}$ children whose parents are both $A_{2}A_{2}$. The reverse is true for mothers, where the children of $A_{1}A_{1}$ mothers all have $-\beta_{i}$, with the exception of $A_{1}A_{1}$ children whose parents are both $A_{1}A_{1}$, and the children of $A_{2}A_{2}$ mothers all have $+\beta_{i}$, with the exception of $A_{2}A_{2}$ children whose parents are both $A_{2}A_{2}$. This clearly shows that when there are indirect parental genetic effects, the phenotypes of the reciprocal heterozygotes will differ, creating the appearance of parent-of-origin effects. Likewise, differences between reciprocal heterozygotes due to parent-of-origin effects will appear as parental genetic effects.

Table 1 also provides the expected probability of the different parent–child combinations within the population (mating frequency), *z*, under RM and Hardy–Weinberg equilibrium, where $(q_{1}+q_{2})=1=(q_{1}+q_{2}{)}^{2}$, as well as the probabilities under AM, $z_{\mathrm{AM}}$, which will be discussed in the next section. The phenotypic values, $Y_{c}$, and expected probabilities, *z*, in Table 1 can be used to calculate the mean and variance contributed by direct, indirect parental genetic, and parent-of-origin effects within the human population under RM. The population mean is given by


(1) $$\mu_{\mathrm{RM}}=\sum_{j}Y_{cj}z_{j}=2q_{2}(\beta_{c}+\beta_{m}+\beta_{f}),$$

where *j* loops over all possible genotypes of the child. The mean is not influenced by the parent-of-origin effects, as they are on average zero. The genetic variance for a single locus is:


(2) $$\begin{array}{rl} V_{\mathrm{RM}} & =\sum_{j}Y_{cj}^{2}z_{j}-\mu_{\mathrm{RM}}^{2}\\ & =2q_{1}q_{2}(\beta_{c}^{2}+\beta_{m}^{2}+\beta_{f}^{2}+\beta_{i}^{2}+\beta_{c}\beta_{m}+\beta_{c}\beta_{f}+\beta_{m}\beta_{i}-\beta_{f}\beta_{i}). \end{array}$$

More details on the calculations are provided in the Supplementary Material.

Equation (2) shows that direct genetic effect genotypic values covary with both maternal and paternal genetic effect genotypic values, which in turn covary with parent-of-origin effect genotypic values, creating a greater degree of potential confounding between all four components than has been previously considered. The additive genetic variance among child’s genotypes, apart from the expected terms due to direct effects ($\beta_{c}^{2}$), also contains components contributed by parental genetic effects ($\beta_{m}^{2}$, $\beta_{f}^{2}$), direct-parental genetic covariance ($\beta_{c}\beta_{m}$, $\beta_{c}\beta_{f}$), parent-of-origin effects ($\beta_{i}^{2}$), and parental-parent-of-origin genetic covariance ($\beta_{i}\beta_{m}$, $\beta_{i}\beta_{f}$). Parent-of-origin effects contribute not only directly but also dependent on their covariance with parental genetic effects, i.e. $\beta_{i}^{2}+\beta_{i}\beta_{m}-\beta_{i}\beta_{f}$, which represents the degree to which loci with parental genetic effects are also imprinted loci in the child. Note the negative sign of $\beta_{i}\beta_{f}$, which is due to the definition of parent-of-origin effects (positive for the maternally inherited allele and negative for the paternal one).

The implications of Equation (2) are that phenotypic variation can be attributable to the single nucleotide polymorphism (SNP) markers of an individual (i.e. SNP heritability $h_{\mathrm{SNP}}^{2}>0$) and marker–phenotype associations can be discovered in population studies, but the underlying pattern of causality can be anything from: (i) the locus does not directly affect the expression of the trait ($\beta_{c}^{2}=0$), but instead only has an effect in one or both of the parents; to (ii) both direct and parental genetic effects may be present at a locus, but the parental effects may cancel out if they covary negatively; or (iii) any combination of terms and their covariance.

### Differences in siblings

The model presented in the previous section can be extended to include genetic effects of a sibling on the child’s phenotype, additionally to direct, indirect parental, and parent-of-origin genetic effects. We consider sibling effects as the influence of the sibling’s genotype on the child’s phenotype, which is mediated through the sibling’s phenotypes. The derivations of population mean and variance including sibling effects ($\beta_{s}$) are given in the Supplementary Material. However, it is more interesting to look at the relationship of the phenotypic and genotypic differences in siblings as the difference in siblings provides an estimate of direct genetics effects unbiased of indirect parental effects (Brumpton et al. 2020; Morris et al. 2020; Young et al. 2022). Typically, sibling analyses do not consider parent-of-origin effects, nor the indirect genetic effects from siblings. Using the phenotypic differences between child and sibling, $\left(Y_{c}-Y_{s}\right)$, and the expected probabilities of the genotypes for the different sibling combinations, which are given in Table 2, we can calculate the expected mean and variance at population level. The total mean is 0, while the variance is


(3) $$V_{s_{\mathrm{RM}}}=\sum_{j}(Y_{cj}-Y_{sj}{)}^{2}z_{j}=2q_{1}q_{2}(\beta_{c}^{2}+\beta_{i}^{2}+\beta_{s}^{2}-2\beta_{c}\beta_{s}),$$

which shows that components due to indirect parental effects do indeed cancel in the difference, but those due to indirect sibling and imprinting effects remain.

**Table 2.** Extension of Table 1 to include siblings and their differences.

$X_{m}$	$X_{f}$	$X_{c}$ $\left(A_{m}A_{f}\right)$	$X_{s}$ $\left(A_{m}A_{f}\right)$	$Y_{c}$					$Y_{s}$					$Y_{c}-Y_{s}$			$f_{z}$
$A_{1}A_{1}$	$A_{1}A_{1}$	$A_{1}A_{1}$	$A_{1}A_{1}$														1
	$A_{1}A_{2}$	$A_{1}A_{1}$	$A_{1}A_{1}$			$+\beta_{f}$					$+\beta_{f}$						$\frac{1}{2}$
			$A_{1}A_{2}$			$+\beta_{f}$		$+\beta_{s}$	$+\beta_{c}$		$+\beta_{f}$	$-\beta_{i}$		$-\beta_{c}$	$+\beta_{i}$	$+\beta_{s}$	$\frac{1}{2}$
		$A_{1}A_{2}$	$A_{1}A_{1}$	$+\beta_{c}$		$+\beta_{f}$	$-\beta_{i}$				$+\beta_{f}$		$+\beta_{s}$	$+\beta_{c}$	$-\beta_{i}$	$-\beta_{s}$	$\frac{1}{2}$
			$A_{1}A_{2}$	$+\beta_{c}$		$+\beta_{f}$	$-\beta_{i}$	$+\beta_{s}$	$+\beta_{c}$		$+\beta_{f}$	$-\beta_{i}$	$+\beta_{s}$				$\frac{1}{2}$
	$A_{2}A_{2}$	$A_{1}A_{2}$	$A_{1}A_{2}$	$+\beta_{c}$		$+2\beta_{f}$	$-\beta_{i}$	$+\beta_{s}$	$+\beta_{c}$		$+2\beta_{f}$	$-\beta_{i}$	$+\beta_{s}$				1
$A_{1}A_{2}$	$A_{1}A_{1}$	$A_{1}A_{1}$	$A_{1}A_{1}$		$+\beta_{m}$					$+\beta_{m}$							$\frac{1}{2}$
			$A_{2}A_{1}$		$+\beta_{m}$			$+\beta_{s}$	$+\beta_{c}$	$+\beta_{m}$		$+\beta_{i}$		$-\beta_{c}$	$-\beta_{i}$	$+\beta_{s}$	$\frac{1}{2}$
		$A_{2}A_{1}$	$A_{1}A_{1}$	$+\beta_{c}$	$+\beta_{m}$		$+\beta_{i}$			$+\beta_{m}$			$+\beta_{s}$	$+\beta_{c}$	$+\beta_{i}$	$-\beta_{s}$	$\frac{1}{2}$
			$A_{2}A_{1}$	$+\beta_{c}$	$+\beta_{m}$		$+\beta_{i}$	$+\beta_{s}$	$+\beta_{c}$	$+\beta_{m}$		$+\beta_{i}$	$+\beta_{s}$				$\frac{1}{2}$
	$A_{1}A_{2}$	$A_{1}A_{1}$	$A_{1}A_{1}$		$+\beta_{m}$	$+\beta_{f}$				$+\beta_{m}$	$+\beta_{f}$						$\frac{1}{4}$
			$A_{1}A_{2}$		$+\beta_{m}$	$+\beta_{f}$		$+\beta_{s}$	$+\beta_{c}$	$+\beta_{m}$	$+\beta_{f}$	$-\beta_{i}$		$-\beta_{c}$	$+\beta_{i}$	$+\beta_{s}$	$\frac{1}{4}$
			$A_{2}A_{1}$		$+\beta_{m}$	$+\beta_{f}$		$+\beta_{s}$	$+\beta_{c}$	$+\beta_{m}$	$+\beta_{f}$	$+\beta_{i}$		$-\beta_{c}$	$-\beta_{i}$	$+\beta_{s}$	$\frac{1}{4}$
			$A_{2}A_{2}$		$+\beta_{m}$	$+\beta_{f}$		$+2\beta_{s}$	$+2\beta_{c}$	$+\beta_{m}$	$+\beta_{f}$			$-2\beta_{c}$		$+2\beta_{s}$	$\frac{1}{4}$
		$A_{1}A_{2}$	$A_{1}A_{1}$	$+\beta_{c}$	$+\beta_{m}$	$+\beta_{f}$	$-\beta_{i}$			$+\beta_{m}$	$+\beta_{f}$		$+\beta_{s}$	$+\beta_{c}$	$-\beta_{i}$	$-\beta_{s}$	$\frac{1}{4}$
			$A_{1}A_{2}$	$+\beta_{c}$	$+\beta_{m}$	$+\beta_{f}$	$-\beta_{i}$	$+\beta_{s}$	$+\beta_{c}$	$+\beta_{m}$	$+\beta_{f}$	$-\beta_{i}$	$+\beta_{s}$				$\frac{1}{4}$
			$A_{2}A_{1}$	$+\beta_{c}$	$+\beta_{m}$	$+\beta_{f}$	$-\beta_{i}$	$+\beta_{s}$	$+\beta_{c}$	$+\beta_{m}$	$+\beta_{f}$	$+\beta_{i}$	$+\beta_{s}$		$-2\beta_{i}$		$\frac{1}{4}$
			$A_{2}A_{2}$	$+\beta_{c}$	$+\beta_{m}$	$+\beta_{f}$	$-\beta_{i}$	$+2\beta_{s}$	$+2\beta_{c}$	$+\beta_{m}$	$+\beta_{f}$		$+\beta_{s}$	$-\beta_{c}$	$-\beta_{i}$	$+\beta_{s}$	$\frac{1}{4}$
		$A_{2}A_{1}$	$A_{1}A_{1}$	$+\beta_{c}$	$+\beta_{m}$	$+\beta_{f}$	$+\beta_{i}$			$+\beta_{m}$	$+\beta_{f}$		$+\beta_{s}$	$+\beta_{c}$	$+\beta_{i}$	$-\beta_{s}$	$\frac{1}{4}$
			$A_{1}A_{2}$	$+\beta_{c}$	$+\beta_{m}$	$+\beta_{f}$	$+\beta_{i}$	$+\beta_{s}$	$+\beta_{c}$	$+\beta_{m}$	$+\beta_{f}$	$-\beta_{i}$	$+\beta_{s}$		$+2\beta_{i}$		$\frac{1}{4}$
			$A_{2}A_{1}$	$+\beta_{c}$	$+\beta_{m}$	$+\beta_{f}$	$+\beta_{i}$	$+\beta_{s}$	$+\beta_{c}$	$+\beta_{m}$	$+\beta_{f}$	$+\beta_{i}$	$+\beta_{s}$				$\frac{1}{4}$
			$A_{2}A_{2}$	$+\beta_{c}$	$+\beta_{m}$	$+\beta_{f}$	$+\beta_{i}$	$+2\beta_{s}$	$+2\beta_{c}$	$+\beta_{m}$	$+\beta_{f}$		$+\beta_{s}$	$-\beta_{c}$	$+\beta_{i}$	$+\beta_{s}$	$\frac{1}{4}$
		$A_{2}A_{2}$	$A_{1}A_{1}$	$+2\beta_{c}$	$+\beta_{m}$	$+\beta_{f}$				$+\beta_{m}$	$+\beta_{f}$		$+2\beta_{s}$	$+2\beta_{c}$		$-2\beta_{s}$	$\frac{1}{4}$
			$A_{1}A_{2}$	$+2\beta_{c}$	$+\beta_{m}$	$+\beta_{f}$		$+\beta_{s}$	$+\beta_{c}$	$+\beta_{m}$	$+\beta_{f}$	$-\beta_{i}$	$+2\beta_{s}$	$+\beta_{c}$	$+\beta_{i}$	$-\beta_{s}$	$\frac{1}{4}$
			$A_{2}A_{1}$	$+2\beta_{c}$	$+\beta_{m}$	$+\beta_{f}$		$+\beta_{s}$	$+\beta_{c}$	$+\beta_{m}$	$+\beta_{f}$	$+\beta_{i}$	$+2\beta_{s}$	$+\beta_{c}$	$-\beta_{i}$	$-\beta_{s}$	$\frac{1}{4}$
			$A_{2}A_{2}$	$+2\beta_{c}$	$+\beta_{m}$	$+\beta_{f}$		$+2\beta_{s}$	$+2\beta_{c}$	$+\beta_{m}$	$+\beta_{f}$		$+2\beta_{s}$				$\frac{1}{4}$
	$A_{2}A_{2}$	$A_{1}A_{2}$	$A_{1}A_{2}$	$+\beta_{c}$	$+\beta_{m}$	$+2\beta_{f}$	$-\beta_{i}$	$+\beta_{s}$	$+\beta_{c}$	$+\beta_{m}$	$+2\beta_{f}$	$-\beta_{i}$	$+\beta_{s}$				$\frac{1}{2}$
			$A_{2}A_{2}$	$+\beta_{c}$	$+\beta_{m}$	$+2\beta_{f}$	$-\beta_{i}$	$+2\beta_{s}$	$+2\beta_{c}$	$+\beta_{m}$	$+2\beta_{f}$		$+\beta_{s}$	$-\beta_{c}$	$-\beta_{i}$	$+\beta_{s}$	$\frac{1}{2}$
		$A_{2}A_{2}$	$A_{1}A_{2}$	$+2\beta_{c}$	$+\beta_{m}$	$+2\beta_{f}$		$+\beta_{s}$	$+\beta_{c}$	$+\beta_{m}$	$+2\beta_{f}$	$-\beta_{i}$	$+2\beta_{s}$	$+\beta_{c}$	$+\beta_{i}$	$-\beta_{s}$	$\frac{1}{2}$
			$A_{2}A_{2}$	$+2\beta_{c}$	$+\beta_{m}$	$+2\beta_{f}$		$+2\beta_{s}$	$+2\beta_{c}$	$+\beta_{m}$	$+2\beta_{f}$		$+2\beta_{s}$				$\frac{1}{2}$
$A_{2}A_{2}$	$A_{1}A_{1}$	$A_{2}A_{1}$	$A_{2}A_{1}$	$+\beta_{c}$	$+2\beta_{m}$		$+\beta_{i}$	$+\beta_{s}$	$+\beta_{c}$	$+2\beta_{m}$		$+\beta_{i}$	$+\beta_{s}$				1
	$A_{1}A_{2}$	$A_{2}A_{1}$	$A_{2}A_{1}$	$+\beta_{c}$	$+2\beta_{m}$	$+\beta_{f}$	$+\beta_{i}$	$+\beta_{s}$	$+\beta_{c}$	$+2\beta_{m}$	$+\beta_{f}$	$+\beta_{i}$	$+\beta_{s}$				$\frac{1}{2}$
			$A_{2}A_{2}$	$+\beta_{c}$	$+2\beta_{m}$	$+\beta_{f}$	$+\beta_{i}$	$+2\beta_{s}$	$+2\beta_{c}$	$+2\beta_{m}$	$+\beta_{f}$		$+\beta_{s}$	$-\beta_{c}$	$+\beta_{i}$	$+\beta_{s}$	$\frac{1}{2}$
		$A_{2}A_{2}$	$A_{2}A_{1}$	$+2\beta_{c}$	$+2\beta_{m}$	$+\beta_{f}$		$+\beta_{s}$	$+\beta_{c}$	$+2\beta_{m}$	$+\beta_{f}$	$+\beta_{i}$	$+2\beta_{s}$	$+\beta_{c}$	$-\beta_{i}$	$-\beta_{s}$	$\frac{1}{2}$
			$A_{2}A_{2}$	$+2\beta_{c}$	$+2\beta_{m}$	$+\beta_{f}$		$+2\beta_{s}$	$+2\beta_{c}$	$+2\beta_{m}$	$+\beta_{f}$		$+2\beta_{s}$				$\frac{1}{2}$
	$A_{2}A_{2}$	$A_{2}A_{2}$	$A_{2}A_{2}$	$+2\beta_{c}$	$+2\beta_{m}$	$+2\beta_{f}$		$+2\beta_{s}$	$+2\beta_{c}$	$+2\beta_{m}$	$+2\beta_{f}$		$+2\beta_{s}$				1

Mating frequencies are given in Table 1 which have to be multiplied with the factor $f_{z}$ to account for the different sibling combinations. Parental heterozygous genotypes are not ordered and are used interchangeably.

Importantly, if there is an indirect effect from the sibling on an individual’s phenotype, the variance increases with the squared sibling’s effects but decreases with the covariance between the individual and its sibling. This results in a confounding of direct and indirect sibling effect genotypic values, which always oppose each other in sign, as shown in Table 2. Thus, any indirect sibling genetic effect will act to alter the variance due to the direct genetic effects. But, it does appear possible to separate parent-of-origin effects within this experimental design under RM.

## Single-locus model under assortative mating

Assorative mating acts to alter the mating frequencies in the population as it increases parental similarity at trait-associated loci, which is reflected in the correlation, *ρ*, among the parents in their alleles. As a consequence of AM, the genotype frequencies are changed, and thus also the probabilities of parents producing offspring of a certain genotype, given as $z_{\mathrm{AM}}$ in Table 1. But the minor allele frequencies in the population remain invariant. The altered offspring frequencies, $z_{\mathrm{AM}}$, are taken from Stark (1976). This modification leaves the population mean unchanged under AM, but it alters the variance.

Under AM, we find that a single locus with direct, indirect parental and parent-of-origin effects makes the following contribution to the genetic variance:


(4) $$\begin{array}{rl} V_{\mathrm{AM}} & =2q_{1}q_{2}(\beta_{c}^{2}+\beta_{i}^{2}+\beta_{m}^{2}+\beta_{f}^{2}+\beta_{c}\beta_{m}+\beta_{c}\beta_{f}+\beta_{m}\beta_{i}-\beta_{f}\beta_{i})\\ & \;+q_{1}q_{2}\rho (\beta_{c}^{2}+2\beta_{m}^{2}+2\beta_{f}^{2}-\beta_{i}^{2}+4\beta_{c}\beta_{m}+4\beta_{c}\beta_{f}+4\beta_{m}\beta_{f})\\ & \;+q_{1}q_{2}\rho^{2}(\beta_{c}^{2}-\beta_{i}^{2}+2\beta_{c}\beta_{m}+2\beta_{c}\beta_{f}-2\beta_{m}\beta_{i}+2\beta_{f}\beta_{i}+4\beta_{m}\beta_{f}). \end{array}$$

The *ρ* term reflects a correlation among mates at causal SNP variants (or those correlated with an underlying causal variant) and is equivalent to the inbreeding coefficient, *F*. Equation (4) shows that the increase in the variance contributed by a locus under AM is dependent upon the relationships among the direct, parental genetic, and parent-of-origin effects.

In contrast, we find that the single-locus genetic variance under AM for the sibling difference is reduced by the parental correlation, *ρ*:


(5) $$V_{s_{\mathrm{AM}}}=2q_{1}q_{2}(1-\rho )(\beta_{c}^{2}+\beta_{i}^{2}+\beta_{s}^{2}-2\beta_{c}\beta_{s}).$$

More details can be found in the Supplementary Material.

## Multilocus model

We now extend our model from a single locus to multiple loci (assuming a polygenic additive genetic model) by summing over all *l* causal variants and taking into account correlations, $k_{ij}$, between loci. The total genetic variance under RM is


(6) $$\begin{array}{rl} V_{{\mathrm{tot}}_{\mathrm{RM}}} & =\sum_{\;j=1}^{l}V_{{\mathrm{RM}}_{j}}+\sum_{i\neq j}k_{ij}\sqrt{V_{{\mathrm{RM}}_{i}}V_{{\mathrm{RM}}_{j}}}\\ & =\sum_{\;j=1}^{l}V_{{\mathrm{RM}}_{j}}+2\sum_{i,j\;:\;i<j}k_{ij}\sqrt{V_{{\mathrm{RM}}_{i}}V_{{\mathrm{RM}}_{j}}}. \end{array}$$

The covariances between loci, given in the second term of Equation (6), are dependent upon the correlations among the causal variants and the concordance in their effects size.

Extending the single-locus model for differences in siblings to multiple loci can be done through Equation (6), substituting $V_{\mathrm{RM}}$ with Equation (3). We can also easily adapt Equation (6) to include AM by substituting $V_{\mathrm{RM}}$ with $V_{\mathrm{AM}}$, or in case of the sibling difference $V_{\mathrm{sAM}}$, where AM-induced changes within a single locus are already taken into account. AM-induced correlations between loci (both cis and trans), on the other hand, will alter the value of the correlation term, $k_{ij}$.

The change in variance due to AM induced correlations between alleles of mates at a locus and also between loci has been shown by previous theory (Crow and Kimura 1970) and empirical research (Yengo et al. 2018). An equilibrium is reached under AM, where there is inflated genetic variance, a larger correlation between relatives for traits driving AM and increased homozygosity at causal loci. To put our work in the context of previous theory (Crow and Kimura 1970), we use a similar approach to Crow and Kimura (1970) to understand the effect of AM on the variance taking into account multiple loci and parental influences. We follow the approach outlined in Chapter 4.7 of Crow and Kimura (1970), where all alleles of *l* causal loci are assumed to have the same frequencies, $q_{1}$ and $q_{2}=1-q_{1}$, and the same effect *α* on the trait under study. In our work, we consider the parental genetic influence as well as direct effects, and thus *α* needs to be replaced, depending upon whether the allele is inherited from the mother or the father. The variance of a single allele from the mother is determined to be $q_{1}q_{2}(\beta_{c}+\beta_{m}{)}^{2}$, assuming that the effect of the allele is either $\left(\beta_{c}+\beta_{m}\right)$ or 0, with frequency $q_{1}$ and $q_{2}$. We temporarily assume that parent-of-origin effects are absent, as the procedure of Crow and Kimura (1970) is based on the variance of single alleles, but we will introduce these effects again later. The mean of the maternal allele is then given by $q_{1}(\beta_{c}+\beta_{m})+q_{2}\cdot 0=q_{1}(\beta_{c}+\beta_{m})$. The variance is calculated as


(7) $$\begin{array}{rl} V(A_{m}) & =q_{1}[(\beta_{c}+\beta_{m})-q_{1}(\beta_{c}+\beta_{m}){]}^{2}+q_{2}[0-q_{1}(\beta_{c}+\beta_{m}){]}^{2}\\ & =q_{1}q_{2}(\beta_{c}+\beta_{m}{)}^{2}. \end{array}$$

Similarly, the variance for one paternal allele is


(8) $$V(A_{f})=q_{1}q_{2}(\beta_{c}+\beta_{f}{)}^{2}.$$

Without any correlations between loci or alleles, the sum of the total variance of an individual, V(X), is just the sum of the variances over all loci, where one allele at each locus is inherited from the mother and one from the father


(9) $$\begin{array}{rl} V(X) & =\sum_{i=1}^{l}(V(A_{m_{i}})+V(A_{\;f_{i}}))\\ & =lq_{1}q_{2}[(\beta_{c}+\beta_{m}{)}^{2}+(\beta_{c}+\beta_{f}{)}^{2}]\\ & =2lq_{1}q_{2}(\beta_{c}^{2}+\frac{1}{2}\beta_{m}^{2}+\frac{1}{2}\beta_{f}^{2}+\beta_{c}\beta_{m}+\beta_{c}\beta_{f}), \end{array}$$

which is the expectation for the additive genetic variance in a randomly mating population in the presence of direct and indirect parental effects.

Through AM, correlations between alleles of mates and between loci are introduced, as shown in Chapter 4.7 of Crow and Kimura (1970). Relevant correlations present in the child are correlations between the alleles at the same locus, denoted *f*; at different loci, denoted *s*; as well as at different loci, but within the same gamete, denoted *k*. These are also displayed in Fig. 1. Due to the correlations, the total variance then changes to


(10) $$V(X)=\sum_{i=1}^{l}(V(A_{m_{i}})+V(A_{\;f_{i}}))+\sum_{i\neq j}{\mathrm{cov}}_{ij},$$

where the covariance term ${\mathrm{cov}}_{ij}$ consists of the following three parts, adapted from Crow and Kimura (1970):

1. The covariance of a pair of $A_{m}$ and $A_{f}$ alleles at the same locus for all *l* pairs is given by $lfq_{1}q_{2}(\beta_{c}+\beta_{m})(\beta_{c}+\beta_{f})$, where *f* represents the correlation between $A_{m}$ and $A_{f}$.
2. The covariance between alleles at different loci, but within the same gamete, is $l(l-1)/2\cdot kq_{1}q_{2}(\beta_{c}+\beta_{m/f})$, where the correlation is denoted with *k*. The covariance differs for maternal and paternal alleles in the parental effect $\beta_{m/f}$.
3. There are l(l−1) pairs of $A_{m}$ and $A_{f}$ alleles at different loci with correlation *s* and covariance $l(l-1)sq_{1}q_{2}(\beta_{c}+\beta_{m})(\beta_{c}+\beta_{f})$.

These three covariance terms all act to inflate the genetic variance, as is expected from AM. However, AM also induces a change in the mating frequencies, which the first covariance term does not explicitly take into account. Rather, it is assumed that the correlation *f* acts in the same way on all genotypes, which is akin to assuming that the mating frequencies are not changed. Thus, while previous theory has established that an equilibrium is reached under AM, where there is inflated genetic variance, a larger correlation between relatives for traits driving AM and increased homozygosity at causal loci (Crow and Kimura 1970), the increased homozygosity is not directly accounted for when calculating the expected increase in genetic variance.

Our single-locus model under AM presented above incorporates changes in the mating frequency under AM, which accounts for the increase in variability at one locus that is induced by *f* (which we denote as *ρ* above). To extend our model to multiple loci, we therefore only need to consider the covariances across loci. Instead of studying the correlations between a pair of alleles, we look at the correlation *k* between a pair of genotypes at different loci, i.e. $(A_{m}A_{\;f}{)}_{i}$ and $(A_{m}A_{\;f}{)}_{j}$. This also enables us to include imprinting effects.

For $k=\mathrm{corr}((A_{m}A_{\;f}{)}_{i},(A_{m}A_{\;f}{)}_{j})$, the covariance for l(l−1) possible pairs is given by $l(l-1)k\sqrt{V_{\mathrm{AM}i}V_{\mathrm{AM}j}}=l(l-1)kV_{\mathrm{AM}}$, using the variance under AM defined in either Equation (4), or Equation (5) for differences in siblings. Correlations induced by AM between loci will thus inflate the variance by the factor l(l−1)k. Extending this to the case where the correlations between the loci are not equal and effects and allele frequencies are not the same for each locus, the covariance changes to $\sum_{i\neq j}k_{ij}\sqrt{V_{AMi}V_{AMj}}$. Depending on the sign of the covariance term, it will either increase or reduce the genetic variance and is thus important to take into account.

In human studies, another common family design is using transmitted and untransmitted parental alleles (Kong et al. 2018). We show how our work can be placed in the context of transmitted and untransmitted alleles in the Supplementary Material.

### Multilocus simulations

We conducted a simple forward-in-time simulation study using realistic linkage disequilibrium (LD) to confirm our theoretical calculations for multiple loci. We create genotypes and phenotypes of 32,000 unrelated individuals based on about 50,000 single nucleotide polymorphism (SNPs) randomly selected from the 1000 genomes project (Auton et al. 2015). These individuals are the parents for the next generation, creating two offspring in each of the 10 generations. We create mate assortment by ordering the phenotypes according to a specified phenotypic correlation, $\rho_{Y}$, which is a simplified and not realistic process. However, our aim was to simply confirm our theory and to demonstrate the directions of the (co)variances and the degree of confounding under AM. More details on the simulation are given in the Supplementary Material.

**Fig. 2.**
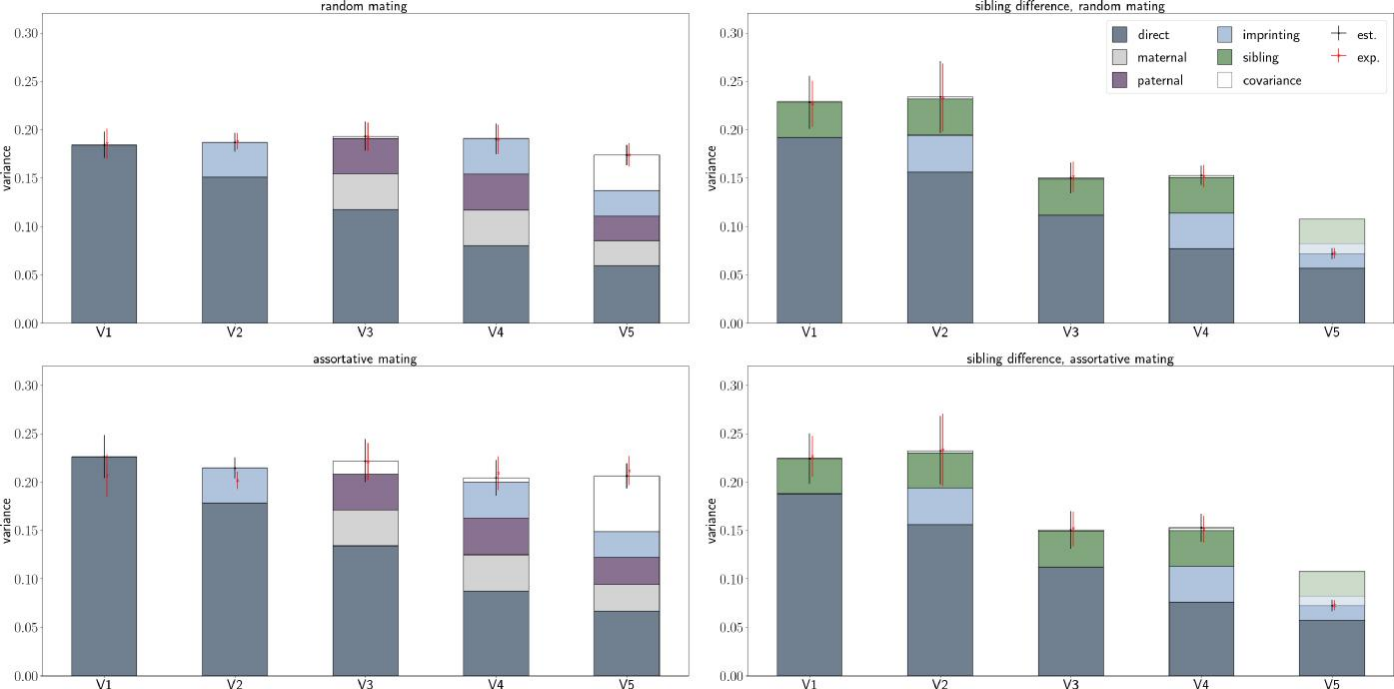
Results of simulation study. Expected theoretical total variance (exp.) calculated using Equation (6), total estimated variance (est.), and estimated variance for each component (represented by the stacked bars) for children (left) and sibling differences (right) under RM (top) and AM ($\rho_{Y}=0.2$, bottom) are shown for various variance–covariance scenarios (V1–V5) for multiple loci. Bars and points represent the mean across 10 simulations, while the uncertainties indicate 2× standard deviations across the 10 simulation scenarios. Note that the direct-sibling covariance is negative and is thus shown opaque, covering parts of the variance.

The left top panel of Fig. 2 shows that the expected theoretical variance matches the estimated variance spread over 1,000 causal loci for a wide range of variance scenarios: (i) only direct effects (V1), (ii) direct and imprinting effects (V2), (iii) direct, maternal and paternal effects (V3), (iv) direct, maternal, paternal, and imprinting effects (V4), and (v) direct, maternal, paternal, and imprinting effects including correlations between them (V5) under RM. As already demonstrated numerically in Equation (2), the population level variance increases when indirect genetic and imprinting effects are present. If there are no parental influences apart from parent-of-origin effects and no sibling effects, then the variance simply increases by $\beta_{i}^{2}$, with no covariance between imprinting and direct effects. If parental genetic effects are also present, covariances then emerge and contribute to the population-level variance. These covariances will be dependent upon the direction and magnitude of the maternal, paternal, and imprinting effects of a given locus. The results given in Fig. 2 clearly show that components of variance cannot be simply separated into independent direct and indirect sources that then simply sum. This highlights again the confounded nature of direct, indirect genetic, and parent-of-origin effects.

Also under AM for a correlation between the parental phenotypes of $\rho_{Y}=0.2$, the estimated and expected theoretical variances match for the different variance scenarios, as shown in the left bottom panel of Fig. 2. If we consider loci with only direct effects (V1), the variance is enlarged only due to the increased homozygosity of causal loci that is expected under AM.

As soon as indirect parental effects exist, the correlation between parental phenotypes generates a genotypic correlation between the parents which in turn creates a covariance term between the maternal and paternal effect genotypic values, $X_{m}\beta_{m}X_{f}\beta_{f}$, as can be clearly seen in V3 comparing the top and bottom left panels of Fig. 2. This term is an important component of variance and can be interpreted as a measure of the similarity of the indirect genetic effects among parents on their children. This suggests that AM on a trait may also create assortment for parental characteristics, where biological parents create similar environments for their children, creating covariance in the indirect genetic effects that may have a large role to play in shaping the population-level variation. Until now this covariance has been generally overlooked. We propose that shared parental effects across traits can also create the cross-trait AM patterns that are observed in the human population (Border et al. 2022).

The right panels of Fig. 2 shows the expected theoretical and estimated variance when modeling the difference between siblings and including indirect sibling effects under RM (top) and AM (bottom). As demonstrated numerically in Equation (3), parental effects do not contribute to the variance as they cancel, although we note that with parental genetic effects the correlation within and among loci under AM alters, which changes the variance as compared to the case when parental genetic effects are absent. However, sibling effects and their covariance with the direct effects do contribute and are indistinguishable from the direct effects here, as we are calculating the difference between siblings. Interestingly, the decrease in variance under AM in case of the sibling difference is more substantial than the increase of variance in trios, as is already expected when comparing Equations (4) and (5).

To show the performance of our theoretical model against various other simulation settings, we added further simulation scenarios to the Supplementary Material, where we vary the number of causal loci and the sample sizes under the realistic variance–covariance scenario V5, and the value of the direct variance under scenario V1 (Supplementary Figs. S1–S3). In this case, when only direct variance and no indirect or imprinting effects exist, the direct variance corresponds to the SNP heritability, $h_{\mathrm{SNP}}^{2}$. When there are indirect and parent-of-origin effects, the definition of $h_{\mathrm{SNP}}^{2}$ as genetic variance over phenotypic variance does not hold any longer.

Moreover, we used the first 20 million basepairs of chromosome 4 to select 52,418 loci to simulate highly correlated markers under the variance–covariance scenarios V1–V5. The results are shown in Supplementary Fig. S4.

### Phenotypic correlation between pairs as function of genomic relationship

**Fig. 3.**
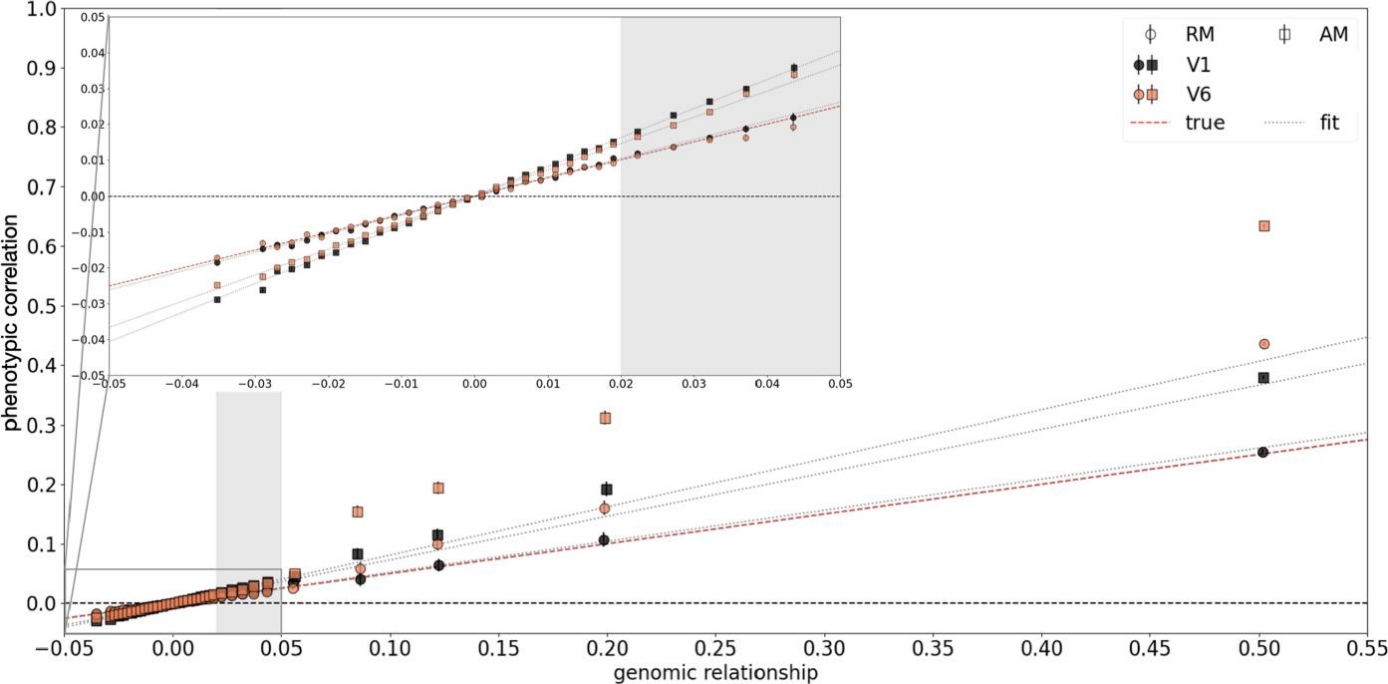
Phenotypic correlation between pairs as function of genomic relationship. Predicted patterns of phenotypic correlation as function of genomic relationship for 64,000 individuals under RM and AM for two different variance scenarios: (V1) only direct effects and (V6) direct effects correlated with indirect maternal and paternal effects. The shaded area indicates the region of sudden increase found in Kemper et al. (2021). The dashed line denoted with “true” in the legend shows the true injected direct variance value, while the dotted lines denoted with “fit” are the slopes fitted to the points in the genomic relationship region between −0.02 and 0.02. The points represent the average of all pairs of individuals found within a genomic relationship bin. Error bars (when visible) show the standard error of Pearson correlations (Bonett 2008).

The phenotypic correlation between pairs of individuals as function of their relatedness, *π*, can be used to estimate the proportion of variance attributable to additive genetic effects (Kemper et al. 2021). Based on the estimates for heritability, $h_{\mathrm{SNP}}=0.5$, and AM, ρ=0.2, for height in the UK Biobank (Kemper et al. 2021), we generate 16,000 families (mother, father, and two children) using our theoretical model. We can thus predict patterns of phenotypic correlations for different degrees of relatedness and compare them against real data in the UK Biobank to gain a better understanding of the effects driving the patterns measured in real data. We study two variance scenarios: (V1) only direct effects, where the heritability under RM corresponds directly to the variance of direct effects; and (V6) direct effects correlated with indirect maternal and paternal effects. More details can be found in the Supplementary Material. Figure 3 shows the generated phenotypic correlation as function of genomic relationship. Under RM, simulating only direct effects, the phenotypic correlation changes linearly from unrelated individuals (π<0.02) to closely related individuals without any change in slope. Under AM, we observe an increase in heritability (i.e. slope) compared to RM and a slight change in slope between unrelated individuals and close relatives. We predict a full-sib phenotypic correlation of 0.379 while the observed value in the UK Biobank for 417,060 individuals for height is 0.528. (Kemper et al. 2021). The estimated slope for close relatives is close to 1 in the UK Biobank. Adding indirect effects in our simulations (V6) shows a similar slope for close relatives under AM. We also find a strong increase in phenotypic correlation from unrelated pairs to close relatives, a similar pattern to what Kemper et al. (2021) observe in the UK Biobank that they attribute to incomplete linkage disequilibrium. Our results however suggest that indirect effects and AM create similar patterns and that indirect effects likely play a role in the change of phenotypic correlation in human height for close relatives.

## Discussion

Here, we provide a quantitative genetics model that includes the influence of indirect maternal and paternal, and parent-of-origin genetic effects on the additive genetic variance. We explicitly derive the influence of correlation of direct genetic, indirect genetic, and epigenetic effects on how AM shapes the population-level genetic variance. Previous theory has established that an equilibrium is reached under AM, where there is inflated genetic variance, a larger correlation between relatives for traits driving AM and increased homozygosity at causal loci (Fisher 1952; Crow and Kimura 1970). But imprinting and indirect genetic effects, alongside their covariances, and the change in mating frequency, have not been considered, when calculating the expected change in genetic variance.

When modeling sibling differences instead of child–mother–father trios, we additionally considered indirect genetic effects from siblings. This design removes indirect parental effects from the variance but alters the variance of direct genetic effects in the presence of indirect sibling genetic effects. This design seems to be able to separate parent-of-origin effects under RM. Under AM, the variances in the trio study design increase, but the variances in the sibling design decrease. Therefore, we suggest that contrasting the estimates across within-family studies may give the upper and lower bounds of the degree to which the components influence the phenotypic variance within the human population.

Our results demonstrate that it is important to control for indirect parental effects when estimating the genetic variance. But even single-locus, marginal estimates common to GWAS from within-family studies are difficult to interpret causally (Veller et al. 2024; Veller and Coop 2024) due to the highly correlated nature of markers and effects. Controlling for the covariances among loci across the genome (both cis- and trans-correlations) when estimating genetic effects would require fitting all variants and all forms of genetic effect (direct, maternal, paternal, and parent-of-origin) jointly. A proposal for this has recently been made within the literature in Krätschmer et al. (2025). We show how covariances depend upon the direction and magnitude of the direct, maternal, paternal, and imprinting effects within and across loci. These covariances contribute substantially to the population-level phenotypic variance, if parental genetic effects are present. In particular, covariance of the parental indirect genetic effects, a measure of the similarity of the indirect genetic effects among parents on their children, may be an important component of variance. We propose that AM on a trait may also create assortment for parental characteristics, where biological parents create similar environments for their children, creating shared parental effects across traits and cross-trait AM (Border et al. 2022).

The separation of imprinting and indirect genetic effects becomes more complex when one considers the underlying potential causal mechanism. Genomic imprinting has been considered as a form of parental effect in the sense that there is parental influence on the genotype–phenotype relationship in the offspring; while others suggest that because the offspring genotype accounts for all of the genetic variance in the offspring trait, imprinting is a form of direct effect as there is no variation explained by the parental genotype. Differentiating the genes in the parental genome that cause imprinting from parental genetic effects and from the genes in the offspring genome that become imprinted is difficult—if not impossible—especially if there are parental loci that control the imprinting state of other loci in the offspring. This complex phenomenon creates variation in offspring traits that is dependent on the combination of parental and offspring genotypes and it is important to recognize that: (i) patterns interpreted as indirect genetic effects can result from genomic imprinting (and vice versa); (ii) it is not straightforward to distinguish parental effects on imprinting from imprinted genes showing parental expression; and (iii) under AM, which is common for many human phenotypes, obtaining accurate estimates will be an even more difficult task.

We have used the terms parent-of-origin and the epigenetic phenomenon of imprinting interchangeably, assuming that parent-of-origin effects are underlain by epigenetic mechanisms, where a parent influences the genotype–phenotype relationship of their children. However, we appreciate that other factors may create parent-of-origin effects in the population (Lawson et al. 2013). Additionally, the *ρ* term used throughout this work reflects a correlation among mates at causal SNP variants (or those correlated with an underlying causal variant). A nonzero *ρ* term can be induced through actively choosing mates similar (or dissimilar) to oneself at specific phenotypic characters, termed direct assortative (disassortative) mating, which creates a phenotypic correlation among parents that translates to a correlation in underlying causal variants. Correlations can also appear through cultural, environmental, or geographical stratification in mating patterns that correlate with trait and trait-associated allele frequency stratification in the population. We do not discriminate between these here as they are likely inseparable in population-level data. Rather, we focus on demonstrating how correlations between mates at causal loci influence the phenotypic variance attributable to the DNA.

Based on the heritability and AM estimates for height in the UK Biobank (Kemper et al. 2021), we generate phenotypic and genotypic data of families using our theoretical model. Thus, we can predict phenotypic correlation patterns of pairs of individuals as function of their genomic relationship under two different variance scenarios under RM and AM. When simulating direct and indirect effects and AM, we find that our predicted slopes follow the ones observed in real data (Kemper et al. 2021), while AM alone is not enough to explain the observed patterns. We therefore suggest that indirect effects play a role in the change of phenotypic correlation for close relatives and that they produce similar patterns to AM. Further studies are needed to better understand how much indirect parental effects shape the phenotypes of their children and if it is possible to disentangle indirect effects from AM.

Here, we ignore dominance, genotype–environment interaction, genotype–environment correlation, epistasis, etc. Future work can incorporate these into our model. Moreover, the framework used here could be combined with mating frequencies expected under different forms of mate choice and thus be used to explore how indirect and epigenetic effects shape phenotypic variation in other systems, for example under sexual selection and directional mate choice.

In summary, population-level variance in human phenotypes is shaped by potentially complex relationships between direct, indirect genetic, and imprinting effects that render their separation and quantification almost impossible, especially when AM occurs within the population. Statistical modeling developments are required if we are to understand the genetic basis of human complex traits in the presence of indirect parental and imprinting effects.

## Supplementary Material

iyag042_Supplementary_Data

## Data Availability

The code used to simulate data can be found at https://github.com/medical-genomics-group/familyMC. Simulations are based on genotype data from 1000 Genomes Project downloaded from https://ftp.1000genomes.ebi. ac.uk/vol1/ftp/release/20130502/. Supplemental material available at GENETICS online.
