# Supplementary material for: A quantitative genetic model for indirect genetic effects and genomic imprinting under random and assortative mating
Source: Genetics. 2026 Feb 12;232(4):iyag042. doi: 10.1093/genetics/iyag042 (PMC13050188; doi:10.1093/genetics/iyag042)
Supplement: iyag042_Supplementary_Data [file iyag042_supplementary_data.pdf]

Supplementary Material

A quantitative genetic model for indirect genetic effects and genomic imprinting under random and assortative mating

Ilse Krätschmer, Matthew R. Robinson

Single-locus model under random mating

We first consider a single autosomal locus with two alleles (A_1 and A_2) with frequencies q_1 and q_2 that can have the following four effects on the child's phenotype: direct (β_c), indirect maternal (β_m), indirect paternal (β_f) and parent-of-origin (imprinting, β_i). The alleles at the locus are ordered, referring first to the maternally inherited allele and second to the paternally inherited one, i.e. $A_m A_f$. The genotypes, X_c, X_m and X_f, are coded as 0, 1 and 2 for A_1A_1 homozygotes, A_1A_2 or A_2A_1 heterozygotes and A_2A_2 homozygotes, respectively. For heterozygous children, the parent-of-origin effect is positive for maternally inherited A_2 and negative for paternally inherited A_2. The possible genotypic and phenotypic values of the child, Y_c, given the genotype of the parents and imprinting are shown in Table 1, where the phenotypic values represent the deviations from the case where both parents and child have A_1A_1 genotypes.

We begin by assuming random mating and Hardy-Weinberg equilibrium, where $(q_1 + q_2) = 1 = (q_1 + q_2)^2$. The mean phenotypic value over the population is calculated as

$$\mu = \sum_j Y_{cj} z_j, \tag{11}$$

where j loops over all possible genotypes of the child, Y_c is the expected phenotype produced by a given genotype and z represents the corresponding mating frequency. The means for the different genotype classes of the child and the total mean are determined to be

$$\mu(A_1A_1) = q_1^2 q_2(\beta_m + \beta_f)\,, \tag{12}$$

$$\mu(A_1A_2) = q_1 q_2(\beta_c - \beta_i + q_2\beta_m + (1 + q_2)\beta_f)\,, \tag{13}$$

$$\mu(A_2A_1) = q_1 q_2(\beta_c + \beta_i + (1 + q_2)\beta_m + q_2\beta_f)\,, \tag{14}$$

$$\mu(A_2A_2) = q_2^2[2\beta_c + (1 + q_2)(\beta_m + \beta_f)]\,, \tag{15}$$

$$\mu = 2q_2(\beta_c + \beta_m + \beta_f)\,. \tag{16}$$

The variance is calculated as

$$V_{RM} = \sum_j Y_{cj}^2 z_j - \mu^2, \tag{17}$$

where

$$\sum_j Y_{cj}^2 z_j = 2q_2(q_2+1)(\beta_c^2+\beta_m^2+\beta_f^2)+2q_1q_2(\beta_i^2+\beta_i\beta_m-\beta_i\beta_f)+2q_2(3q_2+1)(\beta_c\beta_m+\beta_c\beta_f)+8q_2^2\beta_m\beta_f, \tag{18}$$

and results in

$$V_{RM} = 2q_1q_2(\beta_c^2 + \beta_m^2 + \beta_f^2 + \beta_i^2 + \beta_c\beta_m + \beta_c\beta_f + \beta_m\beta_i - \beta_f\beta_i). \tag{19}$$

Including indirect sibling effects

We first extend our single autosomal locus model to the case of siblings where there are the following five effects on the child's phenotype: direct (β_c), indirect maternal (β_m), indirect paternal (β_f), parent-of-origin (imprinting, β_i) and sibling (β_s). The possible genotypic and phenotypic values of the child, Y_c, given the genotype of the parents, sibling and imprinting are shown in Table 2, where the phenotypic values represent the deviations from the case where all parents and children have A_1A_1 genotypes.

We begin by assuming random mating and Hardy-Weinberg equilibrium, where the means for the different genotype classes of the child and the total mean are determined to be

$$\mu(A_1A_1) = q_1^2q_2(\beta_m + \beta_f + \beta_s)\,, \tag{20}$$

$$\mu(A_1A_2) = q_1q_2[\beta_c - \beta_i + q_2\beta_m + (1+q_2)\beta_f + \frac{1}{2}(1+2q_2)\beta_s]\,, \tag{21}$$

$$\mu(A_2A_1) = q_1q_2[\beta_c + \beta_i + (1+q_2)\beta_m + q_2\beta_f + \frac{1}{2}(1+2q_2)\beta_s]\,, \tag{22}$$

$$\mu(A_2A_2) = q_2^2[2\beta_c + (1+q_2)(\beta_m + \beta_f + \beta_s)]\,, \tag{23}$$

$$\mu = 2q_2(\beta_c + \beta_m + \beta_f + \beta_s)\,. \tag{24}$$

The variance is calculated as

$$V_{RM} = \sum_j Y_{cj}^2 z_j - \mu^2, \tag{25}$$

where

$$\begin{aligned}\sum_j Y_{cj}^2 z_j =& 2q_2(q_2+1)(\beta_c^2 + \beta_m^2 + \beta_f^2 + \beta_s^2) + 2q_1q_2(\beta_i^2 + \beta_m\beta_i - \beta_f\beta_i) \\ &+ 8q_2^2\beta_m\beta_f + 2q_2(3q_2+1)(\beta_c\beta_m + \beta_c\beta_f + \beta_c\beta_s + \beta_m\beta_s + \beta_f\beta_s),\end{aligned} \tag{26}$$

and results in

$$V_{RM} = 2q_1q_2(\beta_c^2+\beta_i^2+\beta_m^2+\beta_f^2+\beta_s^2+\beta_c\beta_m+\beta_c\beta_f+\beta_m\beta_i-\beta_f\beta_i+\beta_c\beta_s+\beta_m\beta_s+\beta_f\beta_s). \quad (27)$$

The mean and population-level variance depend on the mating frequency, z, of the parents. We again introduce a correlation ρ between the maternal and paternal alleles which impacts the mating frequencies, z_{AM}, as can be seen in Table 1. The means for the different genotype classes of the child and the total mean are now determined to be

$$\mu(A_1A_1) = q_1q_2(1-\rho)(\beta_m+\beta_f+\beta_s)[q_1+\rho(\frac{1}{2}-q_1)]\,, \quad (28)$$

$$\begin{aligned}\mu(A_1A_2) =& q_1q_2[\beta_c-\beta_i+q_2(1-\rho)\beta_m+(1+q_2+\rho q_1)\beta_f+\frac{1}{2}(1+2q_2+\rho q_1-\rho q_2)\beta_s]\\ &+\frac{1}{2}\rho q_1q_2[(1+\rho)(-\beta_c+\beta_i)+(q_1-q_2)(1-\rho)\beta_m\\ &\qquad -(3+\rho-2q_1+2\rho q_1))\beta_f-2(q_2+\rho q_1)\beta_s],\end{aligned} \quad (29)$$

$$\begin{aligned}\mu(A_2A_1) =& q_1q_2[\beta_c+\beta_i+(1+q_2+\rho q_1)\beta_m+q_2(1-\rho)\beta_f+\frac{1}{2}(1+2q_2+\rho q_1-\rho q_2)\beta_s]\\ &+\frac{1}{2}\rho q_1q_2[(1+\rho)(-\beta_c-\beta_i)-(3+\rho-2q_1+2\rho q_1))\beta_m\\ &\qquad +(q_1-q_2)(1-\rho)\beta_f-2(q_2+\rho q_1)\beta_s],\end{aligned} \quad (30)$$

$$\begin{aligned}\mu(A_2A_2) =& q_2^2[2\beta_c+(1+q_2+\rho q_1)(\beta_m+\beta_f+\beta_s)]\\ &+\rho q_1q_2[(1+\rho)\beta_c+\frac{1}{2}(3+\rho-2q_1+2\rho q_1)(\beta_m+\beta_f+\beta_s)]\,,\end{aligned} \quad (31)$$

$$\mu = 2q_2(\beta_c+\beta_m+\beta_f+\beta_s)\,. \quad (32)$$

The genotypic mean is the same as the one assuming that there are no correlations between the parental genotypes, which is expected as the allele frequencies do not change. The variance is then

$$\begin{aligned}V_{AM} =& 2q_1q_2(\beta_c^2+\beta_i^2+\beta_s^2+\beta_m^2+\beta_f^2+\beta_c\beta_m+\beta_c\beta_f+\beta_m\beta_i-\beta_f\beta_i+\beta_c\beta_s+\beta_m\beta_s+\beta_f\beta_s)\\ &+q_1q_2\rho(\beta_c^2-\beta_i^2+\beta_s^2+2\beta_m^2+2\beta_f^2+4\beta_c\beta_m+4\beta_c\beta_f+4\beta_c\beta_s+4\beta_m\beta_s+4\beta_f\beta_s+4\beta_m\beta_f)\\ &+q_1q_2\rho^2(\beta_c^2-\beta_i^2+\beta_s^2+2\beta_c\beta_m+2\beta_c\beta_f+2\beta_c\beta_s+2\beta_m\beta_s+2\beta_f\beta_s-2\beta_m\beta_i+2\beta_f\beta_i+4\beta_m\beta_f),\end{aligned} \quad (33)$$

using

$$\sum_j Y_j^2 z_j = 2q_2(q_2+1)(\beta_c^2+\beta_m^2+\beta_f^2+\beta_s^2) + 2q_1q_2(\beta_i^2+\beta_m\beta_i-\beta_f\beta_i)$$
$$+ 8q_2^2\beta_m\beta_f + 2q_2(3q_2+1)(\beta_c\beta_m+\beta_c\beta_f+\beta_c\beta_s+\beta_m\beta_s+\beta_f\beta_s)$$
$$+ q_1q_2\rho(\beta_c^2-\beta_i^2+\beta_s^2+2\beta_m^2+2\beta_f^2+4\beta_c\beta_m+4\beta_c\beta_f+4\beta_c\beta_s+4\beta_m\beta_s+4\beta_f\beta_s+4\beta_m\beta_f)$$
$$+ q_1q_2\rho^2(\beta_c^2-\beta_i^2+\beta_s^2+2\beta_c\beta_m+2\beta_c\beta_f+2\beta_c\beta_s+2\beta_m\beta_s+2\beta_f\beta_s-2\beta_m\beta_i+2\beta_f\beta_i+4\beta_m\beta_f). \tag{34}$$

This shows that indirect effects of siblings are also confounded with the direct effects, the parental genetic effects and the imprinting effects. Assortative mating acts to inflate the covariance of the indirect sibling genetic effects and all other components, making a partitioning of variance components intractable.

Modelling the phenotypic difference of siblings

Previous studies[17] have proposed to control for indirect effects by looking at the phenotypic and genetic difference in siblings. Table 2 shows the possible genotypic and phenotypic values of the child, Y_c, and sibling, Y_s, given the genotype of the parents, the respective sibling and imprinting, as well as the phenotypic difference between the offspring, $Y_c - Y_s$.

The means in phenotypic difference for the various genotype classes of the child and the total mean are determined to be

$$\mu(A_1A_1) = q_1^2q_2(-\beta_c+\beta_s)\,, \tag{35}$$
$$\mu(A_1A_2) = \frac{1}{2}q_1q_2[(1-2q_2)(\beta_c-\beta_s)-\beta_i]\,, \tag{36}$$
$$\mu(A_2A_1) = \frac{1}{2}q_1q_2[(1-2q_2)(\beta_c-\beta_s)+\beta_i]\,, \tag{37}$$
$$\mu(A_2A_2) = q_1q_2^2(\beta_c-\beta_s)\,, \tag{38}$$
$$\mu = 0\,. \tag{39}$$

The total mean is 0 as expected when calculating the difference between siblings. Thus, the variance is

$$V_{sRM} = \sum_j (Y_{cj}-Y_{sj})^2 z_j = 2q_1q_2(\beta_c^2+\beta_i^2+\beta_s^2-2\beta_c\beta_s), \tag{40}$$

which shows that indirect parental effects do indeed cancel in the difference, but indirect sibling and imprinting effects remain.

Single-locus model under assortative mating

The expected phenotype of an offspring of a given genotype depends on the probability that it was produced by each of the different possible parental genotypes. Assortative mating (AM) acts to alter the mating frequencies in the population as it increases parental similarity at trait-associated loci which is reflected in the correlation, ρ, among the parents in their alleles. This changes the genotype frequencies, but does not affect the minor allele frequencies in the population. The genotype frequencies under AM are given in Ref.[16]. The means for the different genotype classes of the child and the total mean are determined to be

$$\mu(A_1A_1) = q_1q_2(1-\rho)(\beta_m+\beta_f)[q_1+\rho(\frac{1}{2}-q_1)]\,, \tag{41}$$

$$\begin{aligned}\mu(A_1A_2) =& q_1q_2[\beta_c-\beta_i+q_2(1-\rho)\beta_m+(1+q_2+\rho q_1)\beta_f]\\ &+\frac{1}{2}\rho q_1q_2[(1+\rho)(-\beta_c+\beta_i)+(q_1-q_2)(1-\rho)\beta_m-(3+\rho-2q_1+2\rho q_1))\beta_f],\end{aligned} \tag{42}$$

$$\begin{aligned}\mu(A_2A_1) =& q_1q_2[\beta_c+\beta_i+(1+q_2+\rho q_1)\beta_m+q_2(1-\rho)\beta_f]\\ &+\frac{1}{2}\rho q_1q_2[(1+\rho)(-\beta_c-\beta_i)-(3+\rho-2q_1+2\rho q_1))\beta_m+(q_1-q_2)(1-\rho)\beta_f],\end{aligned} \tag{43}$$

$$\mu(A_2A_2) = q_2^2[2\beta_c+(1+q_2+\rho q_1)(\beta_m+\beta_f)] \tag{44}$$

$$+\rho q_1q_2[(1+\rho)\beta_c+\frac{1}{2}(3+\rho-2q_1+2\rho q_1)(\beta_m+\beta_f)]\,, \tag{45}$$

$$\mu = 2q_2(\beta_c+\beta_m+\beta_f)\,. \tag{46}$$

The genotypic mean is the same as the one assuming that there are no correlations between the parental genotypes, which is expected as the allele frequencies do not change. The variance is then

$$\begin{aligned}V_{AM} =& 2q_1q_2(\beta_c^2+\beta_i^2+\beta_m^2+\beta_f^2+\beta_c\beta_m+\beta_c\beta_f+\beta_m\beta_i-\beta_f\beta_i)\\ &+q_1q_2\rho(\beta_c^2+2\beta_m^2+2\beta_f^2-\beta_i^2+4\beta_c\beta_m+4\beta_c\beta_f+4\beta_m\beta_f)\\ &+q_1q_2\rho^2(\beta_c^2-\beta_i^2+2\beta_c\beta_m+2\beta_c\beta_f-2\beta_m\beta_i+2\beta_f\beta_i+4\beta_m\beta_f),\end{aligned} \tag{47}$$

using

$$\sum_j Y_j^2 z_j =$$
$$2q_2[(q_2+1)(\beta_c^2+\beta_m^2+\beta_f^2)+q_1(\beta_i^2+\beta_m\beta_i-\beta_f\beta_i)+(3q_2+1)(\beta_c\beta_m+\beta_c\beta_f)+4q_2\beta_m\beta_f]$$
$$+q_1q_2\rho(\beta_c^2-\beta_i^2+2\beta_m^2+2\beta_f^2+4\beta_c\beta_m+4\beta_c\beta_f+4\beta_m\beta_f)$$
$$+q_1q_2\rho^2(\beta_c^2-\beta_i^2+2\beta_c\beta_m+2\beta_c\beta_f-2\beta_m\beta_i+2\beta_f\beta_i+4\beta_m\beta_f). \tag{48}$$

All terms are increased under assortative mating with the exception of imprinting which is reduced. Note that there is now an additional correlation term between the parental genetic effects, $\beta_m\beta_f$, that depends on ρ.

Modelling the phenotypic difference in siblings

Extending the model to also include the change in genotype frequencies due to assortative mating, we find

$$\mu(A_1A_1) = q_1q_2(1-\rho)(-\beta_c+\beta_s)[q_1+\rho(\frac{1}{2}-q_1)]\,, \tag{49}$$
$$\mu(A_1A_2) = \frac{1}{2}q_1q_2(1-\rho)[(1-2q_2+\rho-2\rho q_1)(\beta_c-\beta_s)-\beta_i]\,, \tag{50}$$
$$\mu(A_2A_1) = \frac{1}{2}q_1q_2(1-\rho)[(1-2q_2+\rho-2\rho q_1)(\beta_c-\beta_s)+\beta_i]\,, \tag{51}$$
$$\mu(A_2A_2) = q_1q_2(1-\rho)(\beta_c-\beta_s)[q_2-\rho(\frac{1}{2}-q_1)]\,, \tag{52}$$
$$\mu = 0\,. \tag{53}$$

The variance is reduced by the parental correlation, ρ,

$$V_{sAM} = \sum_j (Y_{cj}-Y_{sj})^2 z_{AMj} = 2q_1q_2(1-\rho)(\beta_c^2+\beta_i^2+\beta_s^2-2\beta_c\beta_s) \tag{54}$$

Transmitted and untransmitted parental alleles

We sought to place our work in the wider context of previous research, which has focussed on transmitted and untransmitted alleles[3]. The phenotype of a child, Y_c, that is influenced by direct, indirect maternal, indirect paternal and imprinting effects across l

loci, can be written in terms of transmitted (A_m, A_f) and untransmitted maternal and paternal alleles ($A_{\backslash m}$, $A_{\backslash f}$):

$$\begin{aligned} Y_c &= \sum_{j=1}^{l}(X_{c_j}\beta_{c_j} + X_{m_j}\beta_{m_j} + X_{f_j}\beta_{f_j} + X_{i_j}\beta_{i_j}) + \epsilon \\ &= \sum_{j=1}^{l}[(A_{m_j} + A_{f_j})\beta_{c_j} + (A_{m_j} + A_{\backslash m_j})\beta_{m_j} + (A_{f_j} + A_{\backslash f_j})\beta_{f_j} + (A_{m_j} - A_{f_j})\beta_{i_j}] + \epsilon \\ &= \sum_{j=1}^{l}[A_{m_j}(\beta_{c_j} + \beta_{m_j} + \beta_{i_j}) + A_{f_j}(\beta_{c_j} + \beta_{f_j} - \beta_{i_j}) + A_{\backslash m_j}\beta_{m_j} + A_{\backslash f_j}\beta_{f_j}] + \epsilon, \end{aligned} \tag{55}$$

where ϵ represents the residual error due to other environmental influences. Note that the imprinting effect will have a positive sign if only A_m is transmitted, a negative one if only A_f is transmitted, and will be equal to 0 for all the other cases. Thus, a transmitted allele has direct, indirect and imprinting effects, while an untransmitted allele only has indirect effects on a child's phenotype.

Assuming Hardy-Weinberg equilibrium and random mating, thus that the four alleles are independent, the total variance that each transmitted and untransmitted allele contributes to the trait of a child per locus j is

$$\begin{aligned} V_j &= q_{1_j}q_{2_j}[(\beta_{c_j} + \beta_{m_j} + \beta_{i_j})^2 + (\beta_{c_j} + \beta_{f_j} - \beta_{i_j})^2 + \beta_{m_j}^2 + \beta_{f_j}^2] \\ &= 2q_{1_j}q_{2_j}(\beta_{c_j}^2 + \beta_{i_j}^2 + \beta_{m_j}^2 + \beta_{f_j}^2 + \beta_{c_j}\beta_{m_j} + \beta_{c_j}\beta_{f_j} + \beta_{m_j}\beta_{i_j} - \beta_{f_j}\beta_{i_j}), \end{aligned} \tag{56}$$

where q_{1_j} and q_{2_j} are the allele frequencies. Equation 56 is equivalent to Equation 2.

When effect sizes are estimated in GWAS, a linear relationship between an individual's phenotype and their genotype, i.e. the two transmitted alleles, is assumed, ignoring imprinting effects. Thus, the SNP heritability, defined as the proportion of phenotypic variance among children attributable to the SNP genotypes of the children, would correspond to

$$h_{SNP}^2 = \sum_{j=1}^{l} 2q_{1_j}q_{2_j}(\beta_{c_j}^2 + 0.5\beta_{m_j}^2 + 0.5\beta_{f_j}^2 + \beta_{c_j}\beta_{m_j} + \beta_{c_j}\beta_{f_j}) \tag{57}$$

assuming that all loci are independent. Our results suggest that in reality, it is not known how much the indirect effects contribute to the GWAS estimate unless all the direct and

indirect terms are taken into account, as we do not know the size and magnitude of the covariances between direct and paternal effects, nor do we know if indirect components are actually totally estimated or estimated as part of the residual variance, nor is it clear how the abundant covariances across loci will influence the effect estimates (both cis- and trans-LD). It is also important to note that the variance due to the untransmitted alleles does not equal the total variance of the indirect effects, but only half (at most). Thus, the effects attributed to the untransmitted alleles are in fact not a good estimate of indirect parental effects, as has already been noted by Ref.[13].

Previous work[3] used polygenic risk scores computed from transmitted alleles, $\hat{\boldsymbol{g}}_T = \mathbf{A}_m\hat{\boldsymbol{\beta}}_{GWAS} + \mathbf{A}_f\hat{\boldsymbol{\beta}}_{GWAS}$, and untransmitted ones $\hat{\boldsymbol{g}}_{NT} = \mathbf{A}_{\setminus m}\hat{\boldsymbol{\beta}}_{GWAS} + \mathbf{A}_{\setminus f}\hat{\boldsymbol{\beta}}_{GWAS}$, to separate direct from indirect effects. The effects of transmitted and untransmitted alleles, θ_T and θ_{NT}, are estimated from the linear regression,

$$\mathbf{y} = \theta_T\hat{\boldsymbol{g}}_T + \theta_{NT}\hat{\boldsymbol{g}}_{NT} + \boldsymbol{\epsilon}, \tag{58}$$

where $\mathbf{y}$ is a vector of phenotypic observations and $\mathbf{y}$, $\hat{\boldsymbol{g}}_T$ and $\hat{\boldsymbol{g}}_{NT}$ are all standardized to have mean zero and variance 1. The direct effects of the transmitted polygenic risk score, δ, are then determined by calculating the difference between transmitted and untransmitted effects, $\delta = \theta_T - \theta_{NT}$. The estimated variance accounted for by the direct effect alone is then given by

$$\begin{aligned} R^2_\delta &= (\theta_T - \theta_{NT})^2/\theta_T^2 \\ &= \sigma_C^2/(\sigma_C^2 + 0.5 * \sigma_M^2 + 0.5 * \sigma_F^2), \end{aligned} \tag{59}$$

where σ_C^2 is the variance contributed by the direct effects $\sigma_C^2 = \sum_{j=1}^{l} 2q_{1_j}q_{2_j}\beta_{c_j}^2$, σ_M^2 is the variance contributed by the indirect maternal effects $\sigma_M^2 = \sum_{j=1}^{l} 2q_{1_j}q_{2_j}\beta_{m_j}^2$, and σ_F^2 is the variance contributed by the direct effects $\sigma_F^2 = \sum_{j=1}^{l} 2q_{1_j}q_{2_j}\beta_{f_j}^2$.

Equation 59 makes the following assumptions: (i) $\theta_T = (\sigma_C^2 + 0.5 * \sigma_M^2 + 0.5 * \sigma_F^2)$ and $\theta_{NT} = (0.5 * \sigma_M^2 + 0.5 * \sigma_F^2)$; (ii) loci are independent; (iii) there are no covariances among direct and indirect effects; (iv) imprinting effects are absent; (v) transmitted and untransmitted alleles have the same indirect effects; and (vi) estimation error can be ignored. Note that the estimated variance for direct effects, R^2_δ, in Equation 59 is defined

differently than the direct variance, β_c^2, in Equations 2-5. R_δ^2 is a relative estimate of the direct effects in the transmitted predictor, while β_c^2 is an absolute value and can only be transformed to something similar to R_δ^2 by making an assumption on the heritability of the transmitted alleles.

Forward-in-time simulation

A simple, but realistic simulation (based loosely on Ref.[27]) for parents and children is set up and moved forward in time for a number of generations, $ngen = 10$, to simulate assortative mating. Genotypes of 32,000 unrelated individuals in generation 0 are created with a realistic LD based on 52,310 SNPs. The SNPs were randomly selected across all chromosomes from the 1000 genomes project (`http://ftp.1000genomes.ebi.ac.uk/vol1/ftp/release/20130502/`) using vcfrandomsample (`https://github.com/vcflib/vcflib#vcflib`) to downsample the data to make it more manageable. A pair of individuals produce two offspring each by recombining the parents' haplotypes based on a genetic map (release b37, for example from `https://github.com/odelaneau/shapeit5/tree/main/resources/maps/b37`). The offspring of generation 0 become the parents for the next generation. Parents after generation 0 mate based on their ordered genetic values (which is equivalent to the phenotpyes here) to create assortative mating. The phenotypes for ordering are calculated as:

$$\boldsymbol{Y} = \boldsymbol{X_c\beta_c} + \boldsymbol{X_f\beta_f} + \boldsymbol{X_m\beta_m} + \boldsymbol{X_h\beta_h} \tag{60}$$

To facilitate ordering in the case where only very few variants are causal, noise drawn from a Normal distribution with mean 0 and standard deviation of 0.01 is added to the model. The phenotypes are ordered to introduce a correlation, $\rho = 0.25$, between the parents. This procedure is a simplified version of the unknown realistic process. For the implementation of the AM procedure, see Code availability.

Simulation studies were carried out 10 times each for the following five different

variance-covariance matrices of effects,

$$\boldsymbol{V} = \begin{pmatrix} V_c & V_{cf} & V_{cm} & V_{ci} & V_{cs} \\ V_{fc} & V_f & V_{fm} & V_{fi} & V_{fs} \\ V_{mc} & V_{mf} & V_m & V_{mi} & V_{ms} \\ V_{ic} & V_{if} & V_{im} & V_i & V_{is} \\ V_{sc} & V_{sf} & V_{sm} & V_{si} & V_s \end{pmatrix}.$$

When the indirect effects of siblings are not modelled, the last column and the last row are removed.

1. Scenario 1 represents a covariance matrix with only direct effects and, in case of the sibling difference, sibling effects:

$$\boldsymbol{V_1} = \begin{pmatrix} 0.5 & 0.0 & 0.0 & 0.0 & 0.0 \\ 0.0 & 0.0 & 0.0 & 0.0 & 0.0 \\ 0.0 & 0.0 & 0.0 & 0.0 & 0.0 \\ 0.0 & 0.0 & 0.0 & 0.0 & 0.0 \\ 0.0 & 0.0 & 0.0 & 0.0 & 0.1 \end{pmatrix}. \tag{61}$$

2. In the second scenario, direct as well as parent-of-origin effects and, in case of the sibling difference, sibling effects are contributing to the variance:

$$\boldsymbol{V_2} = \begin{pmatrix} 0.4 & 0.0 & 0.0 & 0.0 & 0.0 \\ 0.0 & 0.0 & 0.0 & 0.0 & 0.0 \\ 0.0 & 0.0 & 0.0 & 0.0 & 0.0 \\ 0.0 & 0.0 & 0.0 & 0.1 & 0.0 \\ 0.0 & 0.0 & 0.0 & 0.0 & 0.1 \end{pmatrix}. \tag{62}$$

3. Scenario 3 assumes direct effects as well as smaller indirect maternal and paternal effects and, in case of the sibling difference, sibling effects:

$$\boldsymbol{V_3} = \begin{pmatrix} 0.3 & 0.0 & 0.0 & 0.0 & 0.0 \\ 0.0 & 0.1 & 0.0 & 0.0 & 0.0 \\ 0.0 & 0.0 & 0.1 & 0.0 & 0.0 \\ 0.0 & 0.0 & 0.0 & 0.0 & 0.0 \\ 0.0 & 0.0 & 0.0 & 0.0 & 0.1 \end{pmatrix}. \tag{63}$$

4. In scenario 4, the total variance of 0.5 is spread over all four genetic components. In the case of the sibling difference, there are additionally sibling effects. There are no

correlations.

$$\boldsymbol{V_4} = \begin{pmatrix} 0.2 & 0.0 & 0.0 & 0.0 & 0.0 \\ 0.0 & 0.1 & 0.0 & 0.0 & 0.0 \\ 0.0 & 0.0 & 0.1 & 0.0 & 0.0 \\ 0.0 & 0.0 & 0.0 & 0.1 & 0.0 \\ 0.0 & 0.0 & 0.0 & 0.0 & 0.1 \end{pmatrix}. \tag{64}$$

5. The last scenario represents a scenario where all effects are correlated:

$$\boldsymbol{V_5} = \begin{pmatrix} 0.15 & 0.05 & 0.05 & \text{-}0.02 & 0.05 \\ 0.05 & 0.07 & 0.05 & \text{-}0.02 & 0.05 \\ 0.05 & 0.05 & 0.07 & \text{-}0.02 & 0.05 \\ \text{-}0.02 & \text{-}0.02 & \text{-}0.02 & 0.07 & 0.00 \\ 0.05 & 0.05 & 0.05 & 0.00 & 0.07 \end{pmatrix} \tag{65}$$

Effects were generated for 1000 markers with a multivariate normal distribution with mean 0 and variance $\mathbf{V}$ scaled by the number of causal markers and randomly assigned to 1000 markers for each simulation. The effects were assumed to be constant across generations.

For the comparison of our theoretical predictions against real-data, we used V1 and the following covariance scenario with the parent-of-origin and sibling effects are always set to 0:

$$\boldsymbol{V_6} = \begin{pmatrix} 0.5 & 0.05 & 0.05 & 0.00 & 0.00 \\ 0.05 & 0.01 & 0.05 & 0.00 & 0.00 \\ 0.05 & 0.05 & 0.01 & 0.00 & 0.00 \\ 0.00 & 0.00 & 0.00 & 0.00 & 0.00 \\ 0.00 & 0.00 & 0.00 & 0.00 & 0.00 \end{pmatrix} \tag{66}$$

Effects for 10,000 markers were generated with a multivariate normal distribution with mean 0 and variance which was scaled by $(2q_1q_2)^{1+\alpha}$ where $\alpha = -0.25$. This dependency of the effects on the minor allele frequency was introduced to get the expected distribution of phenotypic covariance. For this study, residual noise was also added to the sum of genetic components in such way that the phenotypes had unit variance. 16,000 families (parents and two children) are used.

The phenotypic covariance between a pair of individuals denoted with 1 and 2 was calculated as the product of their standardized phenotypes, Y_1Y_2. Their genomic rela-

tionship was defined as $\boldsymbol{X_{c1}}\boldsymbol{X_{c2}}^T/p$, where p=52,310 is the number of total markers used in the simulation.

Supplementary Figures

A quantitative genetic model for indirect genetic effects and genomic imprinting under random and assortative mating

Ilse Krätschmer, Matthew R. Robinson

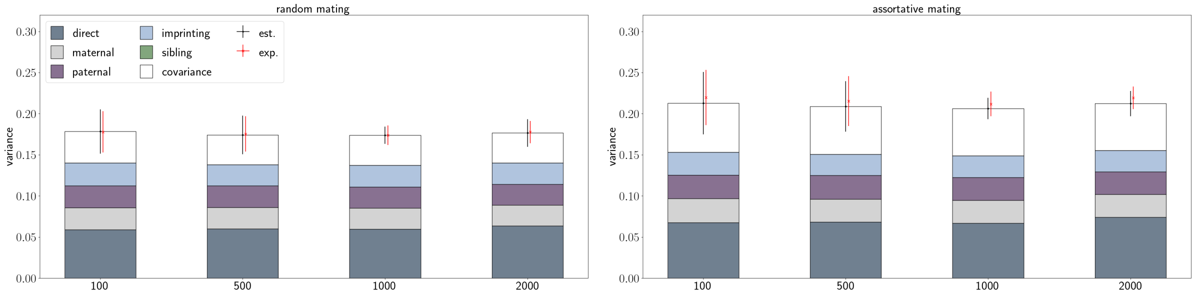

Figure S1: Results of simulation study varying the number of causal loci. Expected theoretical total variance (exp.) calculated using Equation 6, and total estimated variance (est.) and estimated variance and covariance for each component (represented by the stacked bars) for children (left) and sibling differences (right) under random mating (top) and assortative mating ($\rho_Y = 0.2$, bottom) are shown for variance scenario V5 and a different number of causal loci, as indicated on the x-axis. The covariance between loci is added to the direct variance, so that the covariance purely represents the covariance between the direct, indirect and parent-of-origin components. Bars and points represent the mean across 10 simulations, while the uncertainties indicate 2x standard deviations across the 10 simulation scenarios. Note that the direct-sibling covariance is negative and is thus shown opaque, covering parts of the variance.

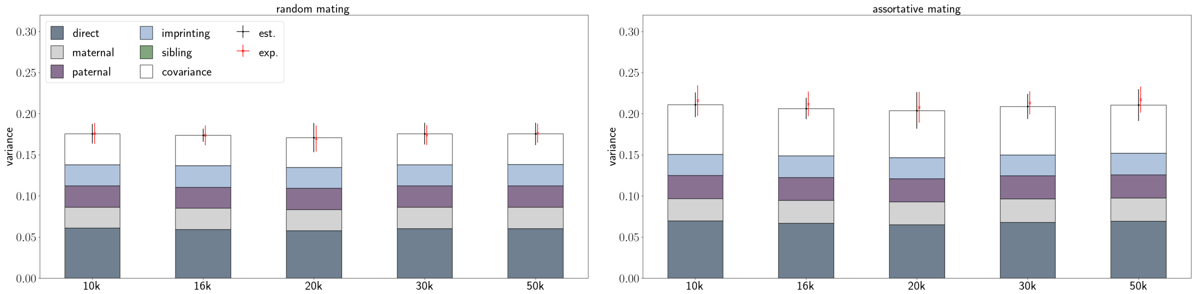

Figure S2: Results of simulation study for different sample sizes. Expected theoretical total variance (exp.) calculated using Equation 6, and total estimated variance (est.) and estimated variance and covariance for each component (represented by the stacked bars) for children (left) and sibling differences (right) under random mating (top) and assortative mating ($\rho_Y = 0.2$, bottom) are shown for variance scenario V5 and varying sample sizes, as indicated on the x-axis. The covariance between loci is added to the direct variance, so that the covariance purely represents the covariance between the direct, indirect and parent-of-origin components. Bars and points represent the mean across 10 simulations, while the uncertainties indicate 2x standard deviations across the 10 simulation scenarios. Note that the direct-sibling covariance is negative and is thus shown opaque, covering parts of the variance.

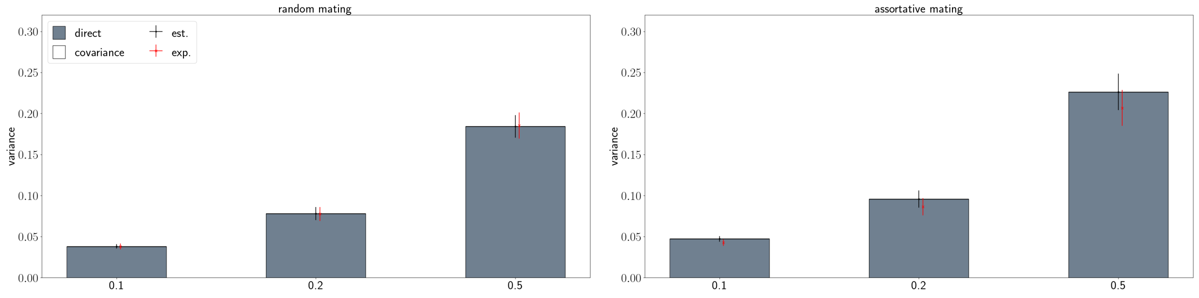

Figure S3: Results of simulation study for different direct variance scenarios. Expected theoretical total variance (exp.) calculated using Equation 6, and total estimated variance (est.) and estimated variance and covariance for each component (represented by the stacked bars) for children (left) and sibling differences (right) under random mating (top) and assortative mating ($\rho_Y = 0.2$, bottom) are shown for variance scenarios, where there is only direct variance with varying variance values, as indicated on the x-axis. The direct variance values correspond to heritability values in this case. Bars and points represent the mean across 10 simulations, while the uncertainties indicate 2x standard deviations across the 10 simulation scenarios. Note that the direct-sibling covariance is negative and is thus shown opaque, covering parts of the variance.

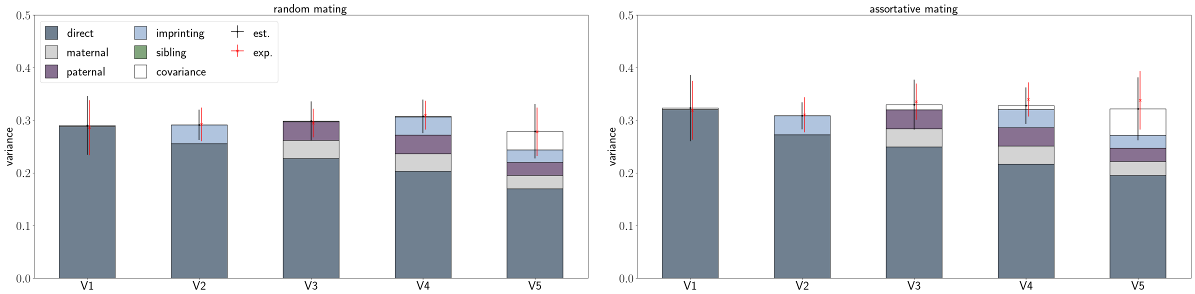

Figure S4: Results of simulation study for 52,418 loci, selected from the first 20 million basepairs of chromosome 4. Expected theoretical total variance (exp.) calculated using Equation 6, and total estimated variance (est.) and estimated variance and covariance for each component (represented by the stacked bars) for children (left) and sibling differences (right) under random mating (top) and assortative mating ($\rho_Y = 0.2$, bottom) are shown for various variance-covariance scenarios (V1-V5) for multiple loci. The covariance between loci is added to the direct variance, so that the covariance purely represents the covariance between the direct, indirect and parent-of-origin components. Bars and points represent the mean across 10 simulations, while the uncertainties indicate 2x standard deviations across the 10 simulation scenarios. Note that the direct-sibling covariance is negative and is thus shown opaque, covering parts of the variance.
